# Improved Automated Current Control for Standard Lamps

**DOI:** 10.6028/jres.099.021

**Published:** 1994

**Authors:** James H. Walker, Ambler Thompson

**Affiliations:** National Institute of Standards and Technology, Gaithersburg, MD 20899-0001

**Keywords:** control, current, lamp, irradiance, radiance, radiometry, standards

## Abstract

As radiometric lamp standards improve, the need to set lamp current to specific values becomes more important. Commercially available power supplies typically provide 12 bit internal digital-to-analog logic which permits current control with a relative expanded uncertainty of about 1 part in 4096, corresponding to an expanded uncertainty of the current of about 2 mA at 8 A (in this paper, expanded uncertainties arc given as 2 standard deviations). For an FEL-type standard spectral irradiance lamp, this corresponds to a spectral irradiance difference of 0.12% at 655 nm. We have developed a technique using 16 bit digital-to-analog conversion which permits current control with a relative expanded uncertainty of about 1 part in 65536, corresponding to an expanded uncertainty of the current of about 0.1 mA at 8 A. This corresponds to a spectral irradiance difference of approximately 0.006% for an FEL lamp at 655 nm. We describe the technique used to achieve this improvement and we show data from a lamp demonstrating the effect of the improvement. We also describe the limitation provided by the uncertainty of the resistance of the current measuring shunt.

## 1. Introduction

Transfer standard lamps (tungsten and tungsten-halogen) for the near ultraviolet, visible, and infrared spectral regions are approximately Planckian thermal sources. This means that the spectral distribution and brightness are functions of the filament temperature and hence the electrical current flowing through the filament. Current changes effect short-wavelength radiation more than long-wavelength radiation. A current change that causes a 0.1% change in radiance at 800 nm causes a 0.2% change at 400 nm. To specify the spectral distribution, the NIST certification of spectral radiance or irradiance of a standard lamp is at a designated current. For radiometric measurements of the highest accuracy it is imperative that the lamp current be known accurately and match the current at which the spectral radiance or irradiance was calibrated.

For spectral radiance and spectral irradiance in the visible, an approximate relationship for determining the change in luminous flux from a lamp due to a current change in the lamp is

$$\phi /\phi_{r}\approx {\left(I/I_{r}\right)}^{6.24},$$

where *ϕ* and *I* are the changed luminous flux and current, respectively, and *ϕ*_r_ and *I*_r_ are the rated values of luminous flux and current, respectively [1] [2]. Using this approximate relationship, an increase in current of 1 mA in a lamp operating at 8.000 A would give (8.001/8.000)^6.24^ = 1.00078 or a change of about 0.078% in luminous flux. By way of comparison, measurements made on our FEL-type standard spectral irradiance lamps operating at 8.000 A give a value of about 0.06% per 1 mA in irradiance at 655 nm. Further, measurements made on standard spectral radiance lamps operating at 40.000 A give a value of about 0.012% per 1 mA in radiance at 655 nm. The approximate relationship gives a value (40.001/40.000)^6.24^ = 1.00016 or a change of about 0.016% in luminous flux.

The current state of the art in spectroradiometry is about one percent relative expanded uncertainty [3] for spectral radiance and spectral irradiance for standard sources in the visible. For tungsten ribbon spectral radiance lamps the component of relative expanded uncertainty attributed to lamp current ranges from 0.01% to 0.14% for gas-filled lamps and from 0.01% to 0.12% for vacuum lamps. For spectral irradiance lamps the component of relative expanded uncertainty attributed to lamp current ranges from 0.01% to 0.08%. These uncertainties would be even larger if the lamps were operated at lower currents to achieve lower spectral radiance and irradiance levels. Depending on the radiance level, the current, and the wavelength being observed, a 1 mA change in lamp current can cause a change in spectral radiance of 0.012% to 0.7% or more. These seemingly small uncertainties will become significant when the overall expanded uncertainty of spectral radiance and irradiance calibrations is reduced by a factor of three or more. Therefore, accurate control of the lamp current is required, especially for measurements in the ultraviolet.

The requirement to control the current in a standard lamp to 1 mA necessitates power supplies with a current stability of 0.01% or less. While many of the currently available commercial power supplies have the required current stability, most use twelve bit digital-to-analog converters to program the current and thus have a current settability of about 4 mA for the operating current of a typical lamp. This fails to meet the requirement. To solve this problem, we have developed a computer-controlled measurement and control system for a constant current power supply. This system utilizes commercially available components to set and maintain the current at the 0.0010% level for both types of standard lamps. We report on the design of this system and the results of spectral radiance measurements using this system with a tungsten vacuum strip lamp.

## 2. Description of Automated Current Control System

**Fig. 1:**
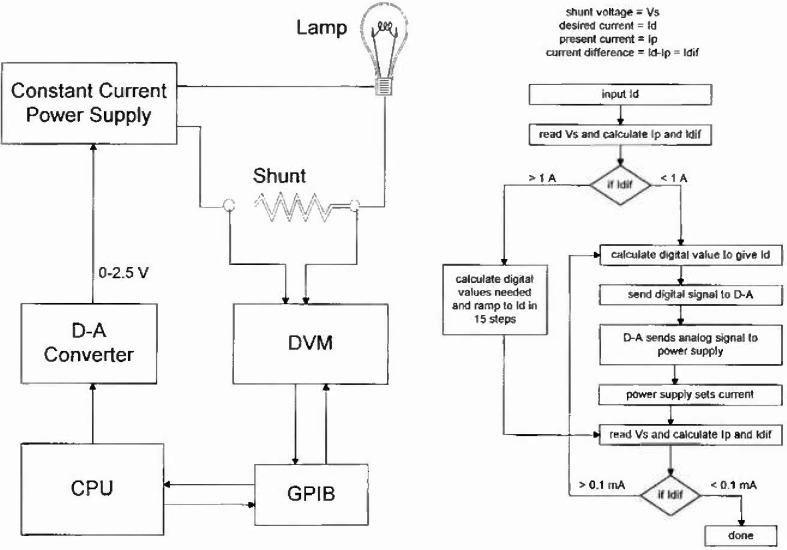
Schematic diagram of the lamp current feedback control system and a programming flowchart.

Figure 1 shows our automated current control system. It is a feedback control system which uses a 16 bit digital-to-analog converter (D-A) located on a board in the computer to produce a control voltage to a voltage controlled constant-current power supply. The power supply current is proportional to this voltage. The load consists of the standard lamp (V40) and a stable shunt resistor. The voltage drop across the shunt resistor is measured by a six-and-a-half-digit digital volt meter (DVM) and the result is acquired by the computer. A data acquisition and control program written in Basic calibrates the circuits and then ramps the current up or down for large current changes or adjusts the control voltage in small amounts to provide accurate constant current operation.

The measurement shunt is nominally rated at 0.01 Ω, 100 A. The shunt was calibrated by the electricity Division at NIST and has a resistance of 0.0099998 Ω with a relative expanded uncertainty of 0.0033% at 8 A. Lamp V40, used as the source for all of the measurements described in this paper, is from a group of specially-constructed, high-stability vacuum lamps of the Quinn-Lee design [4]. This lamp was used because of its extremely small radiance drift rate, on the order of 0.1% per 500 h at 655 nm. The use of this stable lamp permits us to distinguish the effect of small lamp current changes on the spectral radiance. This lamp was calibrated for spectral radiance at a current of 7.600 A in the NIST Facility for Automated Spectroradiometric Calibrations (FASCAL) [5].

A Burr-Brown^1^ PCI-20001C general purpose control board with a Burr-Brown PCI-2006M 16 bit digital-to-analog module was used to produce an output voltage for controlling the current setting of the power supply. This D-A module possesses several programmable output voltage ranges. We used the 0 V to 10 V range. A Hewlett Packard 6030A power supply (0 V to 200 V, 0 A to 17 A, 1000 W, 0.01% line and load regulation) operated in the constant current mode with voltage programming was used to supply the current for the lamp circuit. Since this power supply requires 0 V to 5 V of external voltage control to provide 0 A to 17 A and the operating lamp current was about 7.6 A, an appropriate scaling of voltage output from the D-A board was required. This scaling also limits the voltage output from the D-A board so that the maximum current possible from the power supply does not damage the lamp, and utilizes the full 16 bit programmability of the D-A converter. Initially, an inline 4/1 voltage divider was used to scale the output voltage from the D-A converter. Later it was found that the output could be scaled by bridging of jumpers on the D-A board by a single appropriate resistor. This is the method we used in our applications.

The D-A module is set to a fixed memory address by DIP switches on the control board. A memory address must be used which does not conflict with other components of the computer. The fullscale output range of the D-A converter is set by a jumper on the D-A module. The voltage output of the D-A converter (0 to fullscale voltage) is controlled by writing an integer from −32768 to +32768 to the module memory address using the POKE command. When the computer is turned on, the D-A memory data address is zero, and since this would result in the power supply producing a current of 4 A (half maximum current), we use the autoexec.bat file to run a small program which resets the D-A board to zero voltage output when the computer is turned on. Another subroutine calibrates the power supply current as a function of the signal to the D-A converter with the power supply output short circuited. This information is used by a subroutine which ramps the current up or down to the desired value, within 0.1 mA. The subroutine performs the following steps:

1. The desired lamp current is set by the operator.
2. The present lamp current is read and the difference from the desired current is calculated.
3. If the difference is greater than 1 A, the current is slowly ramped up (or down) to approximately the desired setting by gradually changing the setting of the digital-to-analog converter based on the calibration factor determined for the circuit.
4. If the difference is less than 1 A, a calculation is done using the calibration factor to determine the bit change needed to give the desired current.
5. The lamp current is read again and a new difference is calculated.
6. If necessary, one bit at a time is changed to set the current to within 0.1 mA.

The stability of the D-A output voltage is better than the resolution of one part in 65536 (2^16^) or 0.0015% which corresponds to 0.12 mA at 7.6 A. The gain drift is 25 × 10^−6^/°C or 0.19 mA/°C at 7.6 A. The D-A output voltage replaces the power supply internal program voltage which only has a resolution one part in 4096 (2^12^), corresponding to 4.15 mA at 7.6 A, The use of an external, high-stability shunt to monitor changes in the lamp current improves the sensitivity of the current measurement.

## 3. Results

The radiance measurements described in this paper (except for the calibration of lamp V40) were performed in the NIST Low-Level Spectroradiometric Calibration Facility (LLR). The LLR spectroradiometer uses a 0.67 m McPherson scanning monochromator (f/4.7) that is equipped with a prism predisperser. Since the radiance levels to be measured were relatively high, a silicon detector with an integral amplifier was used to measure the spectral radiance (the LLR normally uses a photomultiplier with a photon counting system). The output of the silicon amplifier was measured by a six-and-one-half-digit DVM. The target viewed by the spectroradiometer for these radiance measurements was 0.6 mm wide by 0.8 mm high, similar to that used in FASCAL. The bandpass of the spectroradiometer was about 2 nm over the wavelength range of 400 nm to 1000 nm.

**Fig. 2:**
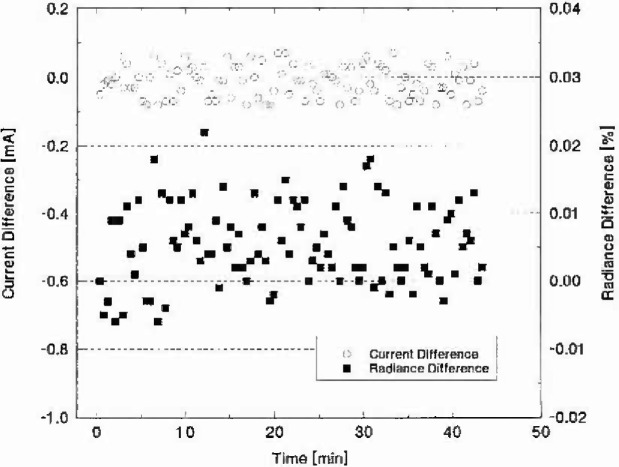
Current and radiance stability as a function of time for lamp V40 operating at 7.6 A. The current was reset to 7.6 A prior to each radiance measurement. The lamp current is expressed as the difference from 7.6 A in milliamperes. The radiance at 900 nm is expressed as the percent difference from the initial radiance measurement.

Continuous measurements were made of the spectral radiance at 900 nm of lamp V40 at 7.600 A over a period of about 43 min. Before each measurement, the lamp current was reset to within 0.1 mA of 7.600 A. Figure 2 shows a plot of the current difference (in mA) and the radiance difference (in%) as a function of time (in minutes). The average current over this period was 7.59999 A with an expanded uncertainty of 0.10 mA. The radiance stability relative expanded uncertainty was 0.012% with no discernable drift over the observed interval. The radiance differences plotted in Fig. 2 are relative to the initial radiance reading.

**Fig. 3:**
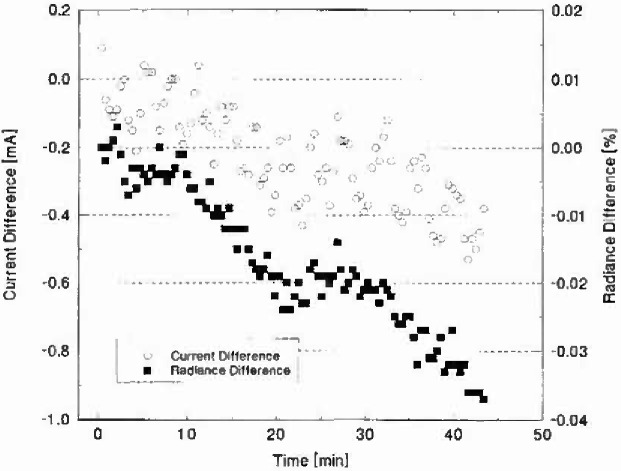
Current and radiance stability as a function of time for lamp V40 operating at 7.6 A. The current was set to 7.6 at the beginning of the time period and the current value was monitored prior to each radiance measurement. The lamp current is expressed as the difference from 7.6 A in milliamperes. The radiance at 900 nm is expressed as the percent difference from the initial radiance measurement.

Additional measurements were carried out under the same conditions as above, except the lamp current was set only at the beginning and the radiance and current were continuously monitored over the observation period. Figure 3 shows the results of these measurements as a plot of the current difference (in mA) and the radiance difference (in%) as a function of time (in minutes). The current decreased 0.4 mA to 0.5 mA with respect to the initial setting over the approximately 43 min time interval. The radiance decreased 0.04% over this time interval. From Fig. 3 it can be seen that a 1 mA change in lamp current corresponds to about a 0.08% change in radiance at 900 nm. The radiance drift would be about twice as large at 400 nm to 500 nm.

**Fig. 4:**
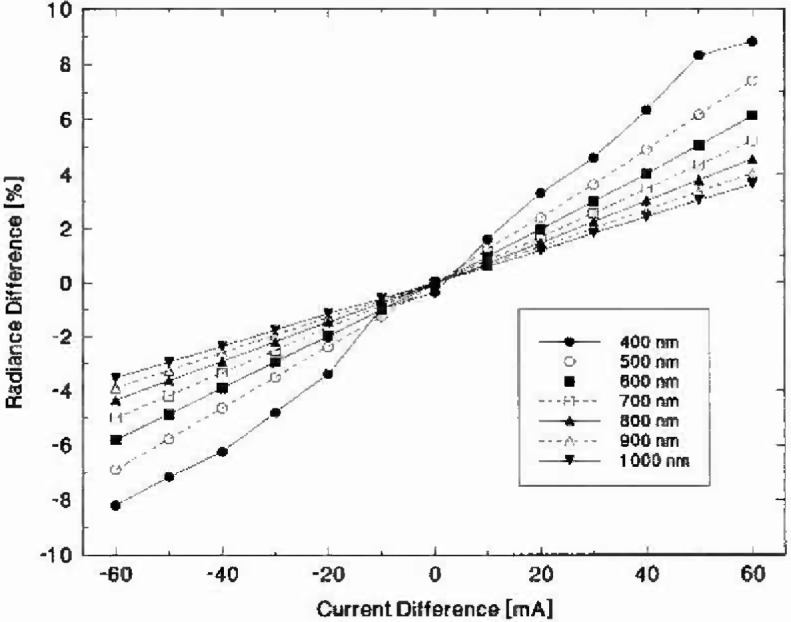
Lamp V40 radiance difference (in percent) as a function of the current difference (in mA from 7.6 A) and wavelength. Wavelengths measured were (400, 500, 600, 700, 800, 900 and 1000) nm.

To examine the wavelength dependence of radiance as a function of lamp current, spectroradiometric scans were made from 400 nm to 1000 nm at 100 nm intervals. The measurement sequence for each wavelength consisted of taking initial measurements at 7.600 A and then varying the lamp current from 7.540 A to 7.660 A in steps of 10 mA and repeating the spectroradiometer scan at the new lamp current setting. A plot of the measured radiance difference (in %) as a function of the measured current difference (in mA) for each of the measured wavelengths is shown in Fig. 4. The two standard deviation radiance repeatability for these measurements was less than 0.01% from 600 nm to 1000 nm, was 0.03% at 500 nm and was 1.30% at 400 nm. In this current range the lamp radiance appears to be directly proportional to the lamp current. A linear fit was applied to the data for each wavelength to determine the slope and these results are summarized in Table 1. The results demonstrate that this slope, which is the rate of change of the lamp radiance with respect to the current, is approximately inversely proportional to the wavelength. This behavior is predicted by the Planck equation. The last column in Table 1 was calculated by taking the slope at 1000 nm and predicting the slope at the other measured wavelengths. The differences between the observed and predicted values for the slope when analyzed in this manner are about 0.1%.

## 4. Discussion

Table 1 shows that the spectral radiance level of lamp V40 would be in error by 0.15% at 400 nm and 0.06% at 1000 nm if the current was in error by 1 mA. If this lamp was operated at 5.000 A, the radiance differences for a 1 mA error would be significantly larger. We have shown that, in order to keep the relative expanded uncertainty in the radiance due to the lamp current below 0.1%, it is necessary to control the current to a fraction of a milliampere. This can be accomplished with our current control technique. However, there is another component of uncertainty that becomes significant when control is done to a fraction of a milliampere. As was stated earlier, the shunt calibration has a relative expanded uncertainty of 0.0033%. For V40 at 7.600 A, this would amount to a 0.25 mA expanded uncertainty in the current setting and a relative expanded uncertainty in the radiance level of 0.015% at 1000 nm and 0.038% at 400 nm. The uncertainty of the shunt resistance is a conservative estimate by the NIST Electricity Division and is due to variation in room temperature causing small changes in the Shunt resistance. Since we use a shunt rated at 100 A and calibrate and use the shunt at 8 A, the expected temperature changes due to power dissipation in the shunt will be small, provided it has a low temperature coefficient. For the manganin type shunt we used, the typical temperature coefficient is about 10 × 10^−6^/°C (or about 0.1 mA/°C at 7.6 A). The expanded uncertainty of the shunt calibration due to temperature fluctuations could be reduced substantially by immersing the shunt in a temperature controlled oil bath. It is also important to use a DVM with sufficient resolution to measure the voltage drop across the shunt. Since we are measuring 7.6 A across a 0.01 Ω shunt, the expected voltage reading is approximately 0.076 V, so a six-and-a half-digit DVM is necessary to achieve the required resolution.

In summary, we have employed an easy-to-use, automated technique for setting and controlling standard lamp current with significantly reduce uncertainty. We have incorporated a 16 bit D-A converter to provide us with the resolution we need to set lamp current to within 0.1 mA and we have used a high stability shunt and a six-and-a-half digit DVM to monitor and feed back the current to the control circuit. These improvements will be more valuable as the expanded uncertainty of spectral radiance and irradiance standards is reduced. In conclusion, it is important to point out that the utilization of standard lamps is dependent on several measurements: the calibration of the lamp for spectral radiance or irradiance and the electrical measurements of resistance and voltage.

## Figures and Tables

**Table 1:** The fitted slope and its uncertainty from the data in Fig. 4 and the predicted slope for lamp V40 operating at 7.6 A. The Wicn approximation of the Planck equation predicts that the slope is inversely proportional to wavelength. The predicted slopes are based upon the observed slope at 1000 nm

Wavelength (nm)	Fitted slope (%/mA)	Standard uncertainty of fitted slope (%/mA)	Predicted slope (%/mA)
400	0.15056	0.00323	0.14933
500	0.11934	0.00081	0.11946
600	0.09933	0.00052	0.09955
700	0.08510	0.00037	0.08533
800	0.07406	0.00035	0.07466
900	0.06588	0.00021	0.06637
1000	0.05973	0.00017	0.05973

## Footnotes

1 Certain commercial equipment, instruments, or materials are identified in this paper to specify adequately the experimental procedure. Such identification docs not imply recommendation or endorsement by the National Institute of Standards and Technology, nor does it imply that the materials or equipment identified are necessarily the best available for the purpose.
